# Prolonged Mechanical Ventilation Assistance Interacts Synergistically with Carbapenem for *Clostridium difficile* Infection in Critically Ill Patients

**DOI:** 10.3390/jcm7080224

**Published:** 2018-08-20

**Authors:** Shyh-Ren Chiang, Chih-Cheng Lai, Chung-Han Ho, Chin-Ming Chen, Chien-Ming Chao, Jhi-Joung Wang, Kuo-Chen Cheng

**Affiliations:** 1 Department of Internal Medicine, Chi Mei Medical Center, 71004 Tainan, Taiwan; chiangsr@gmail.com.tw; 2 Department of General Education, Chia Nan University of Pharmacy and Science, 71710 Tainan, Taiwan; 3 Department of Intensive Care Medicine, Chi Mei Medical Center, 73657 Liouying, Taiwan; dtmed141@gmail.com (C.-C.L.); ccm870958@yahoo.com.tw (C.-M.C.); 4 Department of Medical Research, Chi Mei Medical Center, 71004 Tainan, Taiwan; ho.c.hank@gmail.com (C.-H.H.); 400002@mail.chimei.org.tw (J.-J.W.); 5 Department of Hospital and Health Care Administration, Chia Nan University of Pharmacy and Science, 71710 Tainan, Taiwan; 6 Department of Intensive Care Medicine, Chi Mei Medical Center, 71004 Tainan, Taiwan; chencm3383@gmail.com; 7 Departments of Recreation and Healthcare Management, Chia Nan University of Pharmacy and Science, 71710 Tainan, Taiwan; 8 Department of Safety, Health, and Environmental Engineering, Chung Hwa University of Medical Technology, 71703 Tainan, Taiwan

**Keywords:** mechanical ventilation, interact synergistically, carbapenem, *Clostridium difficile* infection, critically ill patients

## Abstract

Objectives: Interactions between mechanical ventilation (MV) and carbapenem interventions were investigated for the risk of *Clostridium difficile* infection (CDI) in critically ill patients undergoing concurrent carbapenem therapy. Methods: Taiwan’s National Intensive Care Unit Database (NICUD) was used in this analytical, observational, and retrospective study. We analyzed 267,871 intubated patients in subgroups based on the duration of MV support: 7–14 days (*n* = 97,525), 15–21 days (*n* = 52,068), 22–28 days (*n* = 35,264), and 29–60 days (*n* = 70,021). The primary outcome was CDI. Results: Age (>75 years old), prolonged MV assistance (>21 days), carbapenem therapy (>15 days), and high comorbidity scores were identified as independent risk factors for developing CDI. CDI risk increased with longer MV support. The highest rate of CDI was in the MV 29–60 days subgroup (adjusted hazard ratio (AHR) = 2.85; 95% confidence interval (CI) = 1.46–5.58; *p* < 0.02). Moreover, higher CDI rates correlated with the interaction between MV and carbapenem interventions; these CDI risks were increased in the MV 15–21 days (AHR = 2.58; 95% CI = 1.12–5.91) and MV 29–60 days (AHR = 4.63; 95% CI = 1.14–10.03) subgroups than in the non-MV and non-carbapenem subgroups. Conclusions: Both MV support and carbapenem interventions significantly increase the risk that critically ill patients will develop CDI. Moreover, prolonged MV support and carbapenem therapy synergistically induce CDI. These findings provide new insights into the role of MV support in the development of CDI.

## 1. Introduction

*Clostridium difficile* (*C. difficile*) is widely known as a major causal agent of antibiotic-associated colitis [1,2]. Despite current therapeutic options, *C. difficile* infection (CDI) has become one of the most common causes of hospital-acquired infection [3]. A dramatic increase in the incidence and severity over the past decades, especially in critically ill patients in intensive care units (ICUs), has been reported [4,5,6]. Therefore, understanding the pathogenesis and avoiding the risk factors for CDI are critically important for clinicians.

CDI is believed to be precipitated by antimicrobial therapy, the most significant risk factor [7,8], which causes a disruption of the normal colonic microbiota; this disruption predisposes patients to *C. difficile* intestinal colonization [9,10]. Old age, underlying chronic disease, recent hospitalization, gastrointestinal surgeries, and tube feeds were also identified as risk factors associated with *C. difficile* [6,11]. The pathogenesis of CDI is currently attributed to multifactorial disease processes: gut microbial dysbiosis, pathogenic toxin production, and altered host inflammatory responses [9,12,13]. Recent reports highlighted that host immune system interactions with *C. difficile*, rather than the *C. difficile* burden, might be important for determining disease processes and outcomes [14]. Indeed, an excessive host inflammatory response is believed to be crucial for the pathogenesis of CDI [10,13,15,16].

To provide life support for critically ill patients in ICUs, mechanical ventilation (MV) assistance is indispensable. However, clinicians must consider the development of ventilator-associated lung injury (VALI) in patients with MV support [17,18]. Moreover, MV not only initiates lung injury and inflammation, but it also leads to a spillover of the inflammatory mediators from the lungs into the circulation, which propagates a systemic inflammatory response [19,20]. Significant inflammatory responses of the lungs to mechanical stretching can also synergistically interact with a second insult, e.g., exposure to microbial agents or systemic inflammation [21,22] that harms extrapulmonary organs.

MV support and antibiotic therapy might be the two most common interventions in ICUs. However, the role and risk of MV assistance in developing CDI in critically ill patients are largely unclear. MV has been established as a risk factor for the development of CDI in a few studies [23,24], but MV was not a significant risk factor after potential confounders had been adjusted for [5]. Thus, how and why MV support drives CDI is unknown; this also raises another question, i.e., whether MV assistance interacts with antibiotic therapy to provoke CDI in the critically ill patients. We investigated the risk factors for CDI and interactions between MV support and carbapenem therapy in critically ill ICU patients.

## 2. Materials and Methods

### 2.1. Data Source

The NICUD, a sub-database of the Taiwan National Health Insurance Research Database (NHIRD), was our data source. The NICUD contains information on all ICU patients from 1997 to 2013 and is based on the Taiwan National Health Insurance program, which covers almost 100% of the population of Taiwan. Detailed information on inpatients and outpatients was obtained from International Classification of Diseases, Ninth Revision, Clinical Modification (ICD-9-CM) diagnostic codes. All patients listed in the database are de-identified; thus, the Chi Mei Medical Center Institutional Review Board exempted us from having to obtain patient consents.

### 2.2. Patient Selection

**Figure 1:**
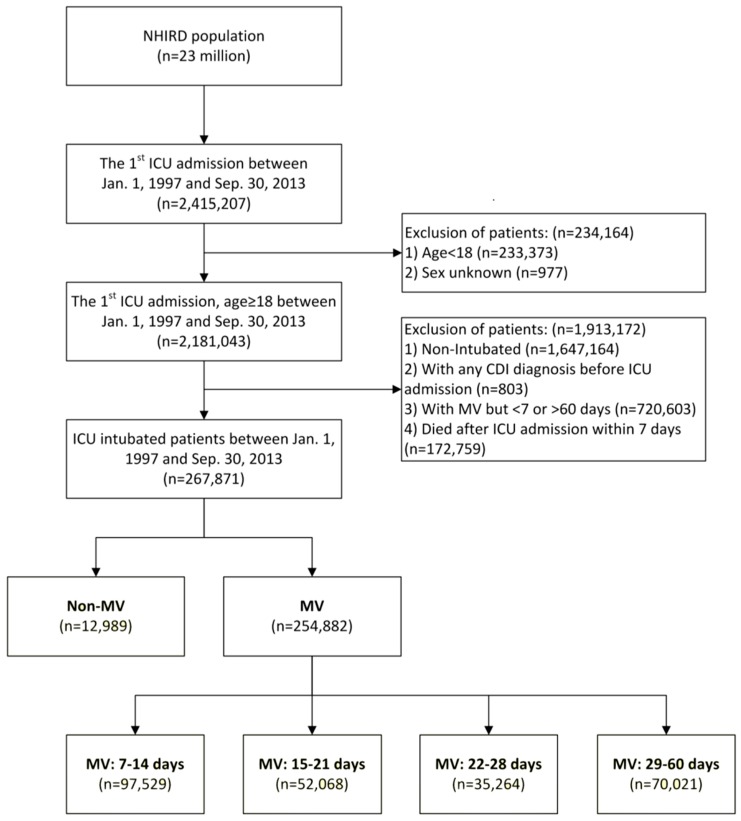
Data from the National Intensive Care Unit Database (NICUD), a sub-database of the Taiwan National Health Insurance Research Database (NHIRD), was used in this study.

Throughout the study period, 2,415,207 patients had been admitted to ICUs. Because most admitted patients were less than 18 years old, of unknown gender, or non-intubated, or CDI was coded before ICU admission, which included MV support less than seven or more than sixty days, or died within seven days after ICU admission, 2,147,336 were excluded. The remaining 267,871 patients were screened, and 254,882 patients with MV support were enrolled (Figure 1).

### 2.3. Measurements

The main event in this study was CDI in ICU intubated patients with or without carbapenem therapy. Follow-up time was calculated from the ICU admission date of the ICU stay to date of death or date of developing CDI within three months after ICU admission. Demographic and clinical characteristics (age, gender, congestive heart failure (CHF) (ICD-9-CM: 398.91, 402.01, 402.11, 402.91, 404.01, 404.03, 404.11, 404.13, 404.91, 404.93, 425.4–425.9, and 428), cerebrovascular accident (CVA) (ICD-9-CM: 436–438), chronic obstructive pulmonary diseases (COPD) (ICD-9-CM: 490–492, and 496), liver disease (ICD-9-CM: 571), chronic kidney disease (CKD) (ICD-9-CM: 585), Charlson Comorbidity Index (CCI) score, days of MV support, and days of carbapenem (imipenem and meropenem)) therapy, were also estimated as potential risk factors for CDI. To examine the effect of MV support and antibiotics therapy on the risk of CDI, the interactions between MV support and antibiotics were also estimated.

### 2.4. Statistical Analysis

A *t* test for continuous variables and a χ^2^ test for categorical variables were used to compare the demographic data, underlying comorbidities, and CDI between patients who had and had not undergone MV. The risk of CDI between these two cohorts was estimated using Cox proportional hazard regression. To consider the possible effects of all confounding factors, the adjusted hazard ratio (AHR) was calculated using Cox regression analysis with the adjustment for multiple variables, including age, gender, MV assistance, antibiotics therapy, comorbidities and CCI scores. Subgroup analyses for antibiotics therapy, MV assistance, duration of MV, and interaction of MV with antibiotics were also performed. Finally, a forest plot was used to determine the risk of CDI for interactions between the duration of MV assistance and antibiotics. Significance was set at *p* < 0.05 (two-tailed). SAS 9.4 (SAS Institute Inc., Cary, NC, USA) was used for all statistical analyses.

## 3. Results

### 3.1. Demographic Data

A total of 267,871 intubated patients in ICUs were enrolled in this study, including MV patients (*n* = 254,882) and controls without MV support (non-MV; *n* = 12,989). MV patients were subgrouped by MV duration: 7–14 days (*n* = 97,525), 15–21 days (*n* = 52,068), 22–28 days (*n* = 35,264), and 29–60 days (*n* = 70,021) (Figure 1).

Individual data in comparable distributions of MV cases and controls (non-MV) by age, sex, carbapenem therapy, ICU days, comorbidities, CCI, and mortality (Table 1). The following variables differed significantly between the MV and non-MV groups: patient age (69.1 vs. 63.7 years old, *p* < 0.0001), female gender (37.9% vs. 43.4%, *p* < 0.0001), carbapenem therapy (30.8% vs. 10.6%, *p* < 0.0001), ICU stay time (11 vs. 2 days, *p* < 0.0001), comorbidity and CCI scores (*p* < 0.0001). The hospital and one-year mortality rates were also higher in the MV group than in the non-MV group (34.0% vs. 32.9%, *p* = 0.0118, and 59.2% vs. 52.7%, *p* < 0.0001, respectively). CDI developed in 435 of 267,871 patients (0.16%); the MV group had a significantly higher rate of CDI than did the non-MV group (0.17% vs. 0.07%, *p* = 0.0069) (Table 1).

The AHRs of CDI were significantly higher with age ≥75 years old (AHR = 1.43; 95% CI = 1.12–1.83; *p* = 0.0041), prolonged MV support (22–28 days: AHR = 2.34, 95% CI = 1.17–4.68, *p* = 0.017, and 29–60 days: AHR = 2.39, 95% CI = 1.21–4.69, *p* = 0.0117), carbapenem therapy more than 15 days (AHR = 1.88; 95% CI = 1.54–2.30; *p* < 0.0001), and high comorbidity scores (CCI 1–2: AHR = 1.62; 95% CI = 1.24–2.10; *p* < 0.0004, and CCI ≥ 3: AHR = 1.65; 95% CI = 1.22–2.24; *p* < 0.0012). These variables were identified as independent risk factors for developing CDI (Table 2).

### 3.2. Analysis of the Risk of Developing CDI with Interactions between MV Support and Carbapenem Therapy

Both carbapenem therapy and MV support were identified as risk factors for developing CDI. Compared with the CDI risk in patients without carbapenem or MV intervention, the AHRs for developing CDI were significantly higher with carbapenem therapy (AHR = 1.92; 95% CI = 1.59–2.32; *p* < 0.0001) and MV support (AHR = 2.19; 95% CI = 1.13–4.24; *p* = 0.0199) (Table 3). Additionally, a progressive increase in CDI risk from the MV 22–28 days subgroup (AHR = 2.72; 95% CI = 1.36–5.44; *p* = 0.0046) to the MV 29–60 days subgroup (AHR = 2.85; 95% CI = 1.46–5.58; *p* = 0.0023) correlated with prolonged MV assistance (>21 days) for developing CDI. Furthermore, patients given carbapenem monotherapy (AHR = 1.99, 95% CI = 0.41–9.60, *p* = 0.3898) or MV support (AHR = 1.94, 95% CI = 0.91–4.11, *p* = 0.0857) had nonsignificantly higher risks for CDI than did the non-MV and carbapenem subgroups. In contrast, there was a significant increase of the risk in the subgroup given both MV and carbapenem interventions (AHR = 3.64; 95% CI = 1.71–7.75; *p* < 0.0008) (Table 3).

### 3.3. Analysis of Synergistic Interactions between the Duration of MV Assistance and Carbapenem Therapy for CDI

**Figure 2:**
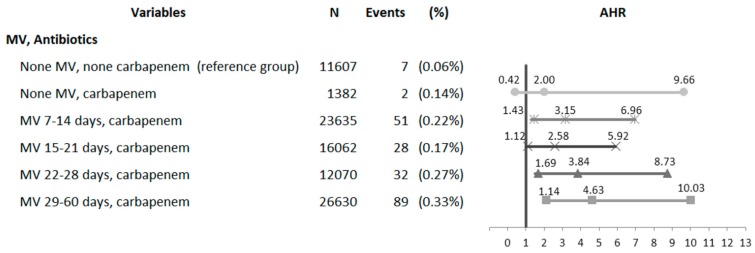
Forest plot of interacted results for the risk of *C. difficile* infection between the mechanical ventilation support and carbapenem therapy in critically ill ICU patients.

A higher but nonsignificant CDI risk correlated with carbapenem monotherapy (Figure 2). However, the CDI risks concurrent with MV and carbapenem interventions were significantly higher in the MV 7–14 days (AHR = 3.15; 95% CI = 1.43–6.96) and MV 15–21 days (AHR = 2.58; 95% CI = 1.12–5.92) subgroups. The CDI rate was positively associated with prolonged MV duration (>21 days) and concurrent carbapenem therapy.

The difference between the 22–28 days MV subgroup (AHR = 3.84; 95% CI = 1.69–8.73) to the 29–60 days MV subgroup (AHR = 4.63; 95% CI = 1.14–10.03) was significant (Figure 2). These results show the risk of developing CDI because of the synergistic interaction between prolonged MV support and concurrent carbapenem therapy.

## 4. Discussion

Our most important finding was that prolonged MV (>21 days) and prolonged carbapenem therapy (>15 days) are independent predictors of CDI. One study [24] reported that the duration of acute MV greatly influenced the development of CDI, and prolonged MV (>3 weeks) was associated with a significantly higher (2–3 times) CDI risk (AHR: 2.72 for MV 22–28 days and AHR: 2.85 for MV 29–60 days) compared with patients without MV. Moreover, after prescribing carbapenem, synergistic interactions between prolonged MV and carbapenem therapy resulted in a significant increase in CDI risk (AHR: 3.84 for MV 22–28 days and AHR: 4.63 for MV 29–60 days). This is the first study to report a synergistic interaction between MV and antibiotics therapy that resulted in an increased risk of CDI. These findings provide novel insights and might support interactions between prolonged MV and antibiotic therapy for the development of CDI.

A dramatic and significantly higher (2–4 times) incidence of CDI was reported in the past decade in North America [25,26]. The reported incidence of CDI in North American and European ICUs is 0.4–4% of the ICU population [27]. We found that 435 of 267,871 (0.16%) critically ill patients developed CDI. This was lower than the previously reported rate (14/1488 (0.9%)) in Taiwan [27]. However, because of the different sample populations and different methodologies used, the incidence rate in the present study might be reasonable.

After a multivariate analysis, elderly patients (>75 years old) (AHR: 1.43, *p* = 0.004) and patients with prolonged MV (22–28 MV days (AHR: 2.34, *p* = 0.015), 29–60 MV days (AHR = 2.39, *p* = 0.0117)), long-duration carbapenem therapy (>15 days (AHR = 1.88, *p* < 0.001)) and a high CCI were at a high risk for developing CDI.

Advanced age is an established risk factor for CDI [28,29]. Indeed, age-related susceptibility to CDI with a 2% increase in the rate every additional year after 18 years old was reported [7]. Multiple interacting factors might be correlated with age in patients with CDI, such as comorbidities [30], polypharmacy interactions [23], progressive immune dysregulation due to old age, and aging accompanied by immune senescence with diminished antibodies against *C. difficile* toxins [31], which leads to the high risk in elderly patients.

Antibiotic interactions are an important risk factor for developing CDI. Because almost any type of antibiotic therapy can lead to this infection [10], carbapenem therapy, the last resort for treating bacterial infections resistant to other available antibiotics, was evaluated in our study. Critically ill patients might be treated with several antibiotics before beginning carbapenem therapy. Thus, patients given carbapenem therapy might have advanced infections and prolonged inflammation.

A cumulative burden of comorbid illness assessment, based on the CCI with scores of 1 (moderate), 2 (severe), and ≥3 (very severe) [32], showed significant differences in the risk for CDI between these three groups (*p* = 0.018). Consistent with other reports, the high CCI score was associated with a high risk of developing CDI and sepsis [33,34,35], which led to CDI after antibiotic therapy.

Both prolonged carbapenem therapy and MV support were identified as independent risk factors for developing CDI. Moreover, a longer duration of MV (>21 days) contributed to a higher CDI risk with carbapenem therapy (AHR: 3.84 in the MV 22–28 days (AHR: 3.84) and MV 29–60 days (AHR: 4.63)) compared with patients without MV and carbapenem interventions. These findings provided important evidence of a synergistic interaction between MV and carbapenem therapy that increases the risk of CDI. The specific mechanisms responsible for the synergistic interactions between prolonged MV and carbapenem merit further investigation and might, in part, contribute to several mechanisms, including: First, critically ill ICU patients with prolonged MV are primarily the elderly with comorbidities and repeated episodes of shock and infection during their ICU stay [36,37]. This condition was associated with persistent inflammation, immunosuppression, and catabolism syndrome that resulted in an increased susceptibility to CDI [38,39]. Second, these patients have more infections with virulent and resistant nosocomial pathogens. Nosocomial pneumonia is the most important infectious disease in patients who require prolonged MV [39]. Prolonged MV patients are more vulnerable to infections with virulent and resistant nosocomial pathogens that require long-term and broad-spectrum antibiotic treatment, which render patients more susceptible to CDI [36,40]. Third, increased permeability and local neutrophilic infiltration of the gut by organ crosstalk is suspected in prolonged MV patients. The release of inflammatory mediators followed by increased neutrophilic infiltration and permeability of the gut are important for developing CDI [41,42,43]. Moreover, MV propagates systemic inflammatory responses, which results in neutrophil recruitment, which, in turn, leads to distal organ damage [44]. Additionally, one study reported that increases in plasma tumor necrosis factor-alpha after MV led to increases in gut permeability [45]. Indeed, a crosstalk between the lungs and the gut during MV was reported [46]. Fourth, cooperation and synergism between transcriptional factors and sensors of innate immunity between MV and CDI are suspected. Synergistic interactions of bacterial products and MV, even in conventional tidal volumes, for lung injury have been reported [47,48]. Indeed, cooperation and synergism for transcriptional factors is currently thought to be the underlying molecular mechanism that causes the combined effect of the two insults [22]. Mitogen-activated protein kinase (MAPK) pathways and nuclear factor kappa B (NF-κB) activation contribute to inflammatory responses in CDI [49]. Interestingly, cell stretching in MV can activate similar signaling pathways (MAPK, NF-κB) as CDI to produce proinflammatory cytokines [22]. Moreover, activating the innate immune sensors, or pattern-recognition receptors (Toll-like receptor family: TLR-2, TLR-4, and TLR-5), is important for developing CDI [16,50]. These TLRs also contribute to the molecular mechanism of barotrauma in MV. Indeed, MV increased the expression of TLR-2 both in the lungs and systemically after Staphylococcus aureus pneumonia was reported in an animal study [51].

### Limitations

Our study has some limitations. First, we identified CDI cases based on diagnostic codes provided by physicians in an administrative database; however, there is a possibility of coding errors and misdiagnoses. Second, some CDI variables, e.g., using proton pump inhibitors or tube feeds, and the burden of C. difficile spores, were not recorded in the claims database; thus, we were unable to assess the possibility of residual confounding factors. Third, we were unable to assess adherence to the prescribed medications because the drug use data were not recorded in the claims database.

## 5. Conclusions

Patient age, carbapenem therapy, high CCI, and prolonged MV were identified as potential risk factors for developing CDI by critically ill ICU patients. Moreover, prolonged MV interacted synergistically with carbapenem therapy to induce CDI. Recognizing the causal relationship between MV and CDI might be important for CDI therapy programs.

## Figures and Tables

**Table 1:** The characteristics of the enrolled patients in this study.

Characteristic	Total (*n* = 267,871)	MV (*n* = 254,882)	Non-MV (*n* = 12,989)	*p*
Age (years): mean ± SD	68.8 ± 16.7	69.1 ± 16.5	63.7 ± 19.6	<0.0001
18–64	87,675 (32.73)	82,120 (32.22)	5555 (42.77)	<0.0001
65–74	61,383 (22.92)	58,616 (23.00)	2767 (21.30)	
≥75	118,813 (44.35)	114,146 (44.78)	4667 (35.93)	
Gender				
Female	102,217 (38.16)	96,574 (37.89)	5643 (43.44)	<0.0001
Male	165,654 (61.84)	158,308 (62.11)	7346 (56.56)	
Carbapenem in ICU ^†^	79,779 (29.78)	78,397 (30.76)	1382 (10.64)	<0.0001
ICU duration (days): median (IQR)	10 (6–17)	11 (6–17)	2 (1–5)	<0.0001
Comorbidities				
Congestive heart failure	15,687 (5.86)	15,037 (5.90)	650 (5.00)	<0.0001
Cerebrovascular accident	27,633 (10.32)	26,331 (10.33)	1302 (10.02)	0.2621
Chronic obstructive pulmonary disease	43,935 (16.40)	42,181 (16.55)	1754 (13.50)	<0.0001
Liver disease	20,097 (7.50)	18,935 (7.43)	1162 (8.95)	<0.0001
Chronic kidney disease	12,706 (4.74)	12,110 (4.75)	596 (4.59)	0.3948
CCI: mean ± SD	1.9 ± 2.2	1.9 ± 2.2	1.7 ± 2.1	<0.0001
0	85,527 (31.93)	80,483 (31.58)	5044 (38.83)	<0.0001
1–2	104,476 (39.00)	99,834 (39.17)	4642 (35.74)	
≥3	77,868 (29.07)	74,565 (29.25)	3303 (25.43)	
Hospital mortality	90,918 (33.94)	86,642 (33.99)	4276 (32.92)	0.0118
1-year mortality	157,846 (58.93)	151,002 (59.24)	6844 (52.69)	<0.0001
*Clostridium difficile* infection ^‡^	435 (0.16)	426 (0.17)	9 (0.07)	0.0069

^†^ Including imipenem and meropenem; ^‡^ within 90 days after ICU admission. All data are expressed as n (%) unless otherwise specified. Categorical variables, expressed as counts and percentages, were analyzed using a χ^2^ test. Variables with a normal distribution are expressed as mean ± standard deviation (SD), and were tested for differences using Student’s *t*-test. Variables not normally distribution are expressed as median and interquartile range (IQR) and differences tested using the Mann-Whitney U test. CCI: (Charlson Comorbidity Index).

**Table 2:** Multivariate analysis for *C. difficile* infection in intubated ICU patients.

Variables	Total	Events	(%)	AHR *	(95% CI)	*p*
**Age (years): mean ± SD**						
18–64	87,675	104	(0.12%)	1.00	(Reference)	-
65–74	61,383	100	(0.16%)	1.20	(0.91–1.59)	0.2001
≥75	118,813	231	(0.19%)	1.43	(1.12–1.83)	0.0041
**Gender**						
Female	102,217	172	(0.17%)	1.00	(Reference)	-
Male	165,654	263	(0.16%)	0.95	(0.78–1.16)	0.6235
**MV Support Duration (days)**						
None	12,989	9	(0.07%)	1.00	(Reference)	-
7–14	97,529	126	(0.13%)	1.63	(0.83–3.20)	0.1595
15–21	52,068	67	(0.13%)	1.52	(0.76–3.06)	0.2377
22–28	35,264	73	(0.21%)	2.34	(1.17–4.68)	0.0168
29–60	70,021	160	(0.23%)	2.39	(1.21–4.69)	0.0117
**Carbapenem Therapy Duration (days)**						
None	188,092	233	(0.12%)	1.00	(Reference)	-
1–7	3678	11	(0.30%)	2.02	(1.10–3.10)	0.0227
8–14	10,616	15	(0.14%)	1.19	(0.72–2.01)	0.5117
≥15	65,485	176	(0.27%)	1.88	(1.54–2.30)	<.0001
**Comorbidities**						
CHF	15,687	35	(0.22%)	1.14	(0.80–1.63)	0.4676
CVA	27,633	53	(0.19%)	0.93	(0.69–1.25)	0.6116
COPD	43,935	79	(0.18%)	0.88	(0.68–1.14)	0.3378
Liver disease	20,097	34	(0.17%)	1.05	(0.73–1.51)	0.7896
CKD	12,706	33	(0.26%)	1.38	(0.95–2.02)	0.0902
**CCI**						
0	85,527	89	(0.10%)	1.00	(Reference)	-
1–2	104,476	192	(0.18%)	1.62	(1.24–2.10)	0.0004
≥3	77,868	154	(0.20%)	1.65	(1.22–2.24)	0.0012

* Adjusted hazard ratio (AHR); adjusted for age, sex, MV support days, duration of carbapenem therapy, comorbidities, and Charlson Comorbidity Index (CCI).

**Table 3:** Subgroup analysis of the interaction between carbapenem therapy and mechanical ventilation support for *C. difficile* infection in critically ill ICU patients.

Variables	*n*	Events	(%)	AHR *	(95% CI)	*p*
**Carbapenem**						
No	188,092	233	(0.12%)	1.00	(Reference)	-
Yes	79,779	202	(0.25%)	1.92	(1.59–2.32)	<0.0001
**Mechanical Ventilation**						
No	12,989	9	(0.07%)	1.00	(Reference)	-
Yes	254,882	426	(0.17%)	2.19	(1.13–4.24)	0.0199
**MV Support Duration (days)**						
None	12,989	9	(0.07%)	1.00	(Reference)	-
7–14	97,529	126	(0.13%)	1.76	(0.90–3.46)	0.1013
15–21	52,068	67	(0.13%)	1.73	(0.86–3.48)	0.1219
22–28	35,264	73	(0.21%)	2.72	(1.36–5.44)	0.0046
29–60	70,021	160	(0.23%)	2.85	(1.46–5.58)	0.0023
**MV, Carbapenem**						
No MV, no carbapenem	11,607	7	(0.06%)	1.00	(Reference)	-
No MV, carbapenem	1382	2	(0.14%)	1.99	(0.41–9.60)	0.3898
MV, no carbapenem	176,485	226	(0.13%)	1.94	(0.91–4.11)	0.0857
MV, carbapenem	78,397	200	(0.26%)	3.64	(1.71–7.75)	0.0008

* Adjusted hazard ratio (AHR); adjusted for carbapenem therapy, mechanical ventilation (MV) support, and interactions between the two events.

## Abbreviations

The following abbreviations are used in this manuscript:

CDI	Clostridium difficile infection
NICUD	National Intensive Care Unit Database
MV	mechanical ventilation
